# Clinical, functional, and laboratory course of children and adolescents with severe asthma after discontinuation of biologics

**DOI:** 10.3389/falgy.2025.1672424

**Published:** 2025-08-22

**Authors:** Valentina Agnese Ferraro, Raimondo Junior Castaldo, Fiorenza Alfier, Margherita Amadi, Chiara Pertoldi, Valentina Tonazzo, Stefania Zanconato, Silvia Carraro

**Affiliations:** Unit of Pediatric Allergy and Respiratory Medicine, Women’s and Children’s Health Department, University Hospital of Padova, Padova, Italy

**Keywords:** severe asthma, children, biologic discontinuation, markers, asthma control

## Abstract

**Background:**

Although the benefits of biologics in severe asthma are well established, the optimal strategy to discontinue therapy remains controversial.

**Aim:**

to evaluate clinical, functional, and laboratory course of children and adolescents with severe asthma after biological therapy withdrawal due to sustained good control. Secondary aim was to identify clinical or inflammatory markers predictive of asthma control after discontinuation.

**Materials and methods:**

this retrospective study included patients 6–19 years with severe asthma followed at the University Hospital of Padua in whom a biologic therapy was discontinued after at least 24 months of treatment. Clinical (GINA, CASI, exacerbations), functional (spirometry), inflammatory (FeNO, IgE, eosinophils), pharmacological (ICS dosage), and quality-of-life (PAQLQ) data were collected over a 24-month follow-up.

**Results:**

twenty-three asthmatic patients (34.8% female) were included. 19 treated with Omalizumab, 3 Dupilumab, and 1 Mepolizumab. At withdrawal, all had well-controlled asthma (GINA and CASI 3). Clinical control scores, spirometry, PAQLQ remained stable overtime. No exacerbation increase was observed. One patient resumed biologic therapy. An increase in eosinophil counts was found in patients classified as not fully controlled at 24 months.

**Conclusions:**

clinical and functional benefit of biologics may persist for up to 24 months after biologic withdrawal. After biologic discontinuation, most children maintained symptom control and good quality of life, suggesting that biologic therapy may be stopped in appropriately selected cases. At the same time a close follow-up, including assessment of clinical control, functional parameter and biomarkers, is needed to promptly identify signs associated with possible loss of control.

## 1 Introduction

Severe asthma refers to asthma that is uncontrolled even when patients consistently follow an optimized treatment with high-dose inhaled steroid together with long acting beta2-agonists (ICS-LABA) and contributing factors are managed, or that worsens when high-dose treatment is decreased (1). The overall severe asthma prevalence in children with asthma is 3% within the studied European populations (2). Add-on therapy for severe asthma includes biologics, i.e., Omalizumab, Dupilumab, Mepolizumab and Tezepelumab (3, 4). These drugs are specifically designed to selectively target molecular pathways involved in the pathogenesis of asthma, allowing treatment to be tailored to the patient's individual inflammatory profile (5).

Together with a positive impact on both asthma clinical control and corticosteroid exposure, biologics appear to have a potential disease-modifying effect, meaning they may influence the natural progression of the disease. This aspect is particularly relevant in paediatric patients, where early intervention could help prevent irreversible airway remodelling and potentially induce asthma remission (6). Recently, the concept of asthma remission has been extensively studied with no universally accepted definition. According to the GINA (Global Initiative For Asthma) Strategy Report 2025 (1), two main categories are recognized: clinical remission, defined as the absence of symptoms and exacerbations for a specific period, and complete (or pathophysiological) remission, which also includes normal lung function, absence of bronchial hyperresponsiveness, and airway inflammation.

Moreover, the investigation of remission characteristics either off-treatment or on-treatment, in particular in patients on biologic therapies, is gaining a growing interest.

Although the benefits of biologics in treating severe asthma are well established (7, 8), the decision to discontinue therapy remains controversial, and standardized guidelines are still lacking. Current GINA 2025 recommendations (1) state that discontinuation of biologic therapy may be considered after at least 12 months of a good clinical response, with well-controlled asthma on medium-dose ICS therapy and, in cases of allergic asthma, in the absence of exposure to known triggers. Indeed, elements like stable lung function, fewer exacerbations, and sustained asthma control have been taken into account before stopping biologics (9–12). Moreover, limited data are available in current literature regarding the persistence of biologic beneficial effects after discontinuation (11, 13).

The aim of this retrospective study was to assess the clinical, functional, and laboratory course of patients with severe asthma who discontinued biologic therapy due to sustained good clinical control. Specifically, we evaluate trends over time in clinical control scores (GINA, Composite Asthma Severity Index CASI, exacerbations), lung function, type 2 inflammation markers (fractional exhaled nitric oxide FeNO, total Immunoglobulin E IgE, blood eosinophils), ICS doses, and quality of life. Secondary aim was to investigate whether early clinical or inflammatory markers can distinguish patients with optimal vs. suboptimal asthma control at 1- and 2-years post-biologic withdrawal.

## 2 Materials and methods

The study was approved by the Ethics Committee of Padova General Hospital (protocol number AOP6195/AO/25 approved 10th April 2025), and all parents, and patients where appropriate, gave their written informed consent to the use of their clinical data for the study.

Patients aged between 6 and 19 years with severe asthma regularly followed at the Pediatric Allergy and Respiratory Unit, Department of Women's and Children's Health, University Hospital of Padua, were included in the study if their biologic therapy had been withdrawn due to sustained good disease control after at least 24 months of treatment. Exclusion criteria was the presence of chronic diseases other than asthma.

Patients were diagnosed with severe asthma if they required medium- to high-dose inhaled corticosteroids (ICSs) combined with a second controller (steps 4 and 5 of the GINA treatment strategy) to control symptoms or if they remained uncontrolled despite this therapy (1). The biologic choice was made in accordance with Italian guidance issued by the relevant authorities.

The clinical charts of included patients were reviewed and the following general information were collected: dates of initiation and discontinuation of biologic therapy, documented allergic sensitizations (skin and/or serological tests), associated comorbidities (such as atopic dermatitis, allergic rhinitis/conjunctivitis, food allergies, obesity, gastroesophageal reflux disease).

In addition, the following data were retrospectively retrieved from routine visits conducted at the time of biologic discontinuation (T0) and after 3 (T1), 6 (T2), 12 (T3), and 24 (T4) months:

• Number of asthma exacerbations (defined as worsening of asthma symptoms treated with systemic steroids >3 days);
• Maintenance therapy and daily dose of inhaled corticosteroids (expressed in mcg/day);
• Symptom control according to Global Initiative for Asthma (GINA) (1) and the Composite Asthma Severity Index (CASI) (14). CASI is a validated multidimensional tool that integrates clinical domains, including symptom frequency, use of controller medications, lung function (FEV₁ % predicted), and exacerbation history. The final score, calculated as the sum of the different domains, increases with poorer asthma control (14);
• Spirometric parameters: FEV₁ (Forced Expiratory Volume in 1 s), FEV₁/FVC (Forced Expiratory Volume in 1 s/Forced Vital Capacity), FEF₂₅–₇₅ (Forced Expiratory Flow at 25%–75% of FVC);
• Perceived quality of life, assessed using the standardized PAQLQ (Paediatric Asthma Quality of Life Questionnaire), which evaluates quality of life in the previous four weeks across 23 items in three domains (emotional function, activity limitations, and symptom perception) using a 7-point Likert scale. The final score, calculated as the mean of the three domains, ranges from 1 (poorest quality of life) to 7 (excellent quality of life);
• Type 2 inflammation biomarkers: blood eosinophils, total IgE, and fractional exhaled nitric oxide (FeNO).

At 12 and 24 months after biologic therapy discontinuation, patients were considered “fully controlled” if they had no exacerbations in the previous 12 months, a GINA score of 0 (i.e., “well controlled”), and a CASI score < 3. Patients not meeting these criteria were classified as “not fully controlled.”

Spirometry was performed using a 10-liter bell spirometer (Biomedin, Padua, Italy). At least three spirometric maneuvers were completed, with at least two reproducible maneuvers required for each test. The most accurate FVC and FEV1 of the three maneuvers were considered for data analysis. All spirometric values were analyzed using Z-score according to reference values of the Global Lung Function Initiative powered by European Respiratory Society (15, 16).

FeNO measurement was performed using the NIOX VERO® device, and values were expressed in parts per billion (ppb).

### 2.1 Statistical analysis

The collected data were anonymized and organized within a Microsoft Excel spreadsheet. A descriptive analysis was used with the use of mean with standard deviation or the median with the corresponding interquartile range, depending on the distribution. Regarding inferential analysis, group comparisons were carried out using the student t-test for normally distributed data and the Mann–Whitney test for non-parametric data. The comparison of ICS dosages over time was performed using the Kruskal–Wallis test. A *p*-value of less than 0.05 was considered statistically significant.

## 3 Results

### 3.1 Study population

Twenty-three patients with severe asthma were included in this study, of whom 8 (34.8%) were female. 19 patients had been treated with Omalizumab, 3 with Dupilumab, and 1 with Mepolizumab, and such drugs, after a mean treatment duration of 3.04 years (SD 0.66), had been discontinued because of good disease control, based on clinicians' judgment. At the time of the last administration of biologic therapy the median age of the participants was 15.1 years (IQR 14.5–17.1). All included patients had at least one allergic sensitization to inhaled allergens, proved by skin tests and/or *in vitro* tests, while 39% of the patients showed atopic comorbidities.

At the time of inclusion in this retrospective study, 13 out of the 23 enrolled patients had undergone their last evaluation 24 months after discontinuation of the drug, 7 patients 12 months after discontinuation of the drug, 3 patients 6 months after discontinuation of the drug. Notably, two patients were not evaluated beyond 12 months after discontinuing Omalizumab, being lost to follow-up. Additionally, one patient was not evaluated beyond 6 months after stopping Omalizumab, as the biologic was restarted 9 months after discontinuation due to worsening of asthma, with two acute exacerbations requiring systemic steroids (between 6 and 9 months) and a decline in asthma control according to GINA and CASI scores.

### 3.2 Symptoms control, lung function, quality of life and biomarkers

Clinical, functional, laboratory variables, and quality of life scores are described in Table 1.

**Table 1:** Clinical, functional, laboratory variables, and quality of life scores in children with severe asthma at the time of biologic discontinuation (T0), and after 3 (T1), 6 (T2), 12 (T3), and 24 months (T4).

	T0	T1	T2	T3	T4	T3 vs. T0 p	T4 vs. T0 p
Patients, *n*	23	23	23	20	13		
Patients with at least one course of corticosteroids in the previous 12 months, *n* (%)	2 (8,7%)	—	—	3 (15%)	2 (15.4%)	.08	.29
GINA score: -well controlled, *n* (%)	23 (100)	23 (100)	19 (82.6)	20 (100)	11 (84.6)	—	—
-partly controlled, *n* (%)	0 (0)	0 (0)	4 (17.4)	0 (0)	1 (7.7)	—	—
-uncontrolled, *n* (%)	0 (0)	0 (0)	0 (0)	0 (0)	1 (7.7)	—	—
CASI score, median (IQR)	3 (3–4)	3 (3–4)	3 (3–5)	3 (2.25–3)	3 (3–4)	.080	.570
FEV_1_ (% pred), mean (SD)	93.4 (9.2)	94.0 (10.4)	93.5 (12.1)	93.6 (10.8)	95.7 (12.8)	—	—
FEV_1_ (z-score), mean (SD)	−0.54 (0.78)	−0.50 (0.88)	−0.45 (1.06)	−0.53 (0.89)	−0,13 (1,06)	.848	.149
FEV1/FVC (ratio), mean (SD)	0.87 (0.08)	0.88 (0.09)	0.88 (0.10)	0.89 (0.08)	0,87 (0.08)	—	—
FEV_1_/FVC (z-score), mean (SD)	0.02 (1.32)	0.33 (1.51)	0.35 (1.81)	0.37 (1.19)	0,13 (1.23)	.430	.931
FEF_25−75_ (% pred), mean (SD)	89.1 (19.5)	98.43 (27.73)	92.9 (24.3)	94.4 (18.5)	97 (21.6)	—	—
FEF_25−75_ (z-score), mean (SD)	−0.54 (0.89)	−0.14 (1.24)	−0.39 (1.15)	−0.28 (0.84)	−0,19 (0.95)	.587	.326
PAQLQ total score, median (IQR)	6.86 (6.73–7.0)	—	—	6.88 (6.74–6.98)	6.93 (6.85–7.0)	.850	.999
PAQLQ symptoms, median (IQR)	6.70 (6.50–7.00)	—	—	6.80 (6.55–7.00)	7.0 (6.90–7.00)	.668	.367
PAQLQ activities, median (IQR)	7.00 (6.60–7.00)	—	—	7.00 (6.60–7.00)	6.80 (6.70–7.00)	.929	.375
PAQLQ emotions, median (IQR)	7.0 (7.0–7.0)	—	—	7.0 (7.0–7.0)	7.0 (7.0–7.0)	.992	.999
Blood Total IgE (kU/L), median (IQR)	—	695 [IQR 223.5–814.5]	—	153 [110–320.75]	—	<.001*	—
Blood eosinophils (cells/mm3), median (IQR)	—	280 [IQR 225–360]	—	330 [IQR 227.5–522.5]	—	.336*	—
FeNO (ppb), median (IQR)	—	40 [IQR 30–66.8]	—	64 [50.8–98.8]	—	.014	—
Remission of asthma: - Clinical, *n* (%) - Pathophysiological, *n* (%)	—	23 (100) 2 (8.7)	19 (82.6) 3 (13)	17 (85) 1 (5)	11 (84.6) 1 (7.7)	—	—

Data are expressed as *n* (%), median (1st quartile; 3rd quartile), or mean (± standard deviation, SD). — =tests not included (as for study design or because it is not computable). * = T3 vs. T1 (as for study design). CASI = Composite Asthma Severity Index; FEV₁ = Forced Expiratory Volume in 1 s); FEV₁/FVC = Forced Expiratory Volume in 1 s/Forced Vital Capacity; FEF₂₅–₇₅ = Forced Expiratory Flow at 25%–75% of FVC; PAQLQ = Paediatric Asthma Quality of Life Questionnaire; IgE = Immunoglobulin E; FeNO = fractional exhaled nitric oxide; clinical remission = patients with well-controlled asthma and without exacerbations; pathophysiological remission = patients with clinical remission and normal lung function and absence of airway inflammation (values of FeNO ≤ 25 ppb, normal blood eosinophils and IgE level).

At the time of biologic discontinuation, all patients showed good clinical control according to GINA score (well controlled in all cases) and CASI score (median value 3, IQR 3−4). Only 2 patients at T0 had required at least one course of systemic corticosteroids in the previous 12 months, with no statistically significant increase in this percentage at subsequent timepoints (T0: 8.7%; T3: 15%; T4: 15.4%; T3 vs. T0 *p* = 0.08; T4 vs. T0 *p* = 0.29).

**Figure 1:**
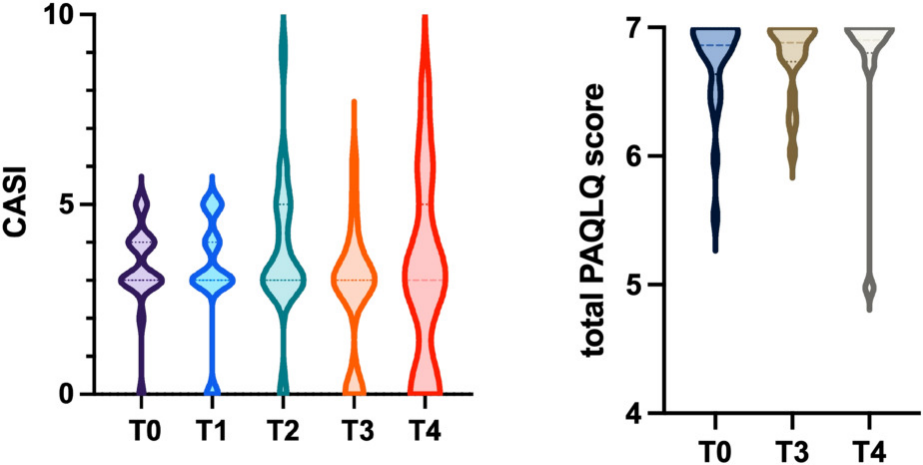
Violin plot showing the distribution of CASI scores at follow-up evaluations. Violin plot showing the distribution of total PAQLQ scores at follow-up assessments. The dashed lines represent the median, first quartile, and third quartile.

Regarding GINA scores during follow-up, the majority of patients maintained well-controlled asthma (score = 0) at all timepoints. At the 6-month assessment, 4 patients were classified as partially controlled and 0 as uncontrolled, while at 24 months, 1 patient was classified as partially controlled and 1 as uncontrolled. However, these variations did not reach statistical significance. The CASI score also remained low and stable over time, with no statistically significant differences observed across the various follow-up assessments (Table 1 and Figure 1).

Baseline spirometric values (T0) were within normal limits and showed no statistically significant changes over the entire follow-up period, including comparisons between T3 and T0 and between T4 and T0 (*p* > 0.05) (Table 1).

At the time of biologic treatment discontinuation (T0), the median total PAQLQ score was 6.9, indicating an excellent health-related quality of life. Over the 1- and 2-year follow-up period (Table 1), no statistically significant changes were observed in the overall PAQLQ score (Figure 1), nor in the scores of the three specific domains assessed: emotional function, activity limitation, and symptom perception.

In the biomarker analysis, total blood IgE, blood eosinophil counts, and FeNO were measured at different time points. Blood total IgE levels 3 months after discontinuation were significantly higher than at 12 months [IgE: T1 median 695 KU/L (IQR 223.5–814.5); T3 median 153 KU/L (110–320.75); *p* < 0.001], while blood eosinophil and FeNO levels showed an opposite trend with higher levels at 12 months [Eosinophils: T1 median 280/mm^3^ (IQR 225–360); T3 median 330/mm^3^ (IQR 227.5–522.5); *p* = 0.336] [FeNO: T1 median 40 ppb (IQR 30–66.8); T3 median 64 ppb (50.8–98.8); *p* = 0.014]. We could not compare values at 24 months because of the small number of patients evaluated at T4, so far.

During the 24 month follow up, the majority of patients met the criteria for clinical remission, whereas only very few patients also fulfilled the criteria for pathophysiological remission (see Table 1).

### 3.3 Maintenance therapy during follow-up

**Figure 2:**
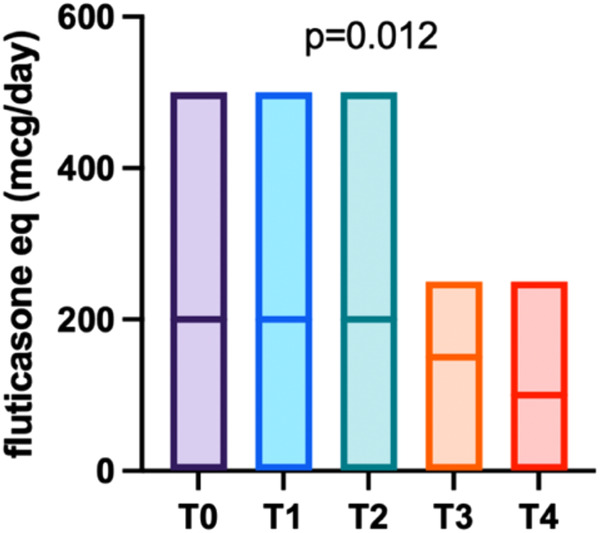
Trend of fluticasone-equivalent dosage over the follow-up period, with the median value represented by a horizontal bar.

During the follow-up period after biologic discontinuation (T0), asthma maintenance therapy based on ICS, possibly in combination with long-acting beta-2 agonists, was adjusted by the treating physicians, mainly according to asthma control. As shown in Figure 2, at T0 patients had a median daily ICS dose of 200 mcg/day (fluticasone equivalent) (IQR 200–250), which significantly decreased over time (*p* = 0.012), reaching a median of 100 mcg/day (IQR 0–250) at 24 months post-discontinuation.

### 3.4 Biomarkers and clinical scores after treatment withdrawal

Taking into consideration the follow-up evaluations at 12 and 24 months (T3 and T4), two groups of patients were compared: those in whom the disease was fully controlled and those with not fully controlled disease. Asthma was considered fully controlled if there were no exacerbations in the previous year, CASI score was 3 or less, and the patient was well controlled according to GINA score. Based on these parameters, 16 out of 20 patients at T3 and 9 out of 13 patients at T4 were classified as fully controlled.

At T3, patients who were not fully controlled had a higher median CASI score at the time of treatment discontinuation compared to fully controlled patients (4.5 vs. 3, *p* = 0.007), as well as after 3 months (5 vs. 3, *p* = 0.027) and 6 months (4.5 vs. 3, *p* = 0.05). No differences were found in the other parameters analysed (eosinophil count, total IgE levels, and GINA score). At T4, patients who were not fully controlled showed significantly higher blood eosinophil levels at 3 and 12 months after treatment discontinuation compared to those who were fully controlled (360/mm^3^ vs. 270/mm^3^, *p* = 0.0448; 665/mm^3^ vs. 260/mm^3^, *p* = 0.02, respectively). No statistically significant differences were found regarding the other biomarkers (total IgE and FeNO) or the GINA and CASI scores.

### 3.5 Patients who dropped out of the study

Of the 23 patients enrolled in the study, 3 were lost to follow-up. Two of them were monitored up to 12 months after treatment discontinuation and were then lost to clinical follow-up, as they did not attend the scheduled evaluations. The third patient, an 18.7-year-old adolescent, restarted omalizumab treatment 9 months after discontinuation due to worsening asthma. This included three acute exacerbations treated with systemic corticosteroids (none requiring hospitalization), deterioration in asthma control with CASI score raised to 9 (from 3 at T0) and GINA score changed from well controlled at T0 to uncontrolled, and a decline in quality of life with a PAQLQ total score of 4.13 (activities 3.6, symptoms 3.8 and emotions 5) (compared to total score of 6.93 at T0). Based on these findings, it was decided to resume Omalizumab therapy.

Data available at the time of discontinuation (T0) did not allow prediction of clinical deterioration in the following months. However, analysis of biomarkers collected 3 months after discontinuation (T1) showed elevated FeNO levels (105 ppb) and increased total serum IgE (812 KU/L) and blood eosinophil counts remained within the normal range (220 cells/μl). Notably, IgE levels had increased by 230% compared to pre-treatment values.

## 4 Discussion

Our data demonstrate a sustained clinical and functional benefit of biologics in patients with severe asthma, even after treatment discontinuation due to persistent good disease control. Over the 12–24 months after biologic discontinuation, children exhibited no increase in asthma exacerbations, maintained good symptom control as assessed by GINA and CASI score, and preserved quality of life according to PAQLQ score. Only one adolescent resumed biologic therapy due to asthma worsening, suggesting that dependence on biologic therapy may be limited to a minority of children with severe allergic asthma.

In our cohort, biologic therapy was discontinued after a period of sustained clinical stability, with a mean treatment duration of 3.04 years (SD 0.66) prior to withdrawal. The decision to stop biologic therapy was made in accordance with GINA 2025 recommendations (1), which suggest that discontinuation of biologic treatment may be considered after at least 12 months of a good clinical response, with well-controlled asthma on medium-dose ICS, and in the absence of exposure to known triggers. The mean treatment duration observed in our cohort aligns with available data in the scientific literature: in the study by Baena-Cagnani et al. (12), the biologic therapy is maintained for 12 months, whereas in the study by Molimard et al. (17), the mean duration of treatment increases to 22.7 months. In the research conducted by Deschildre et al. (10), treatment duration ranges from a minimum of 24 months to a maximum of 48 months. Finally, in the study by Humbert et al. (9), the median treatment duration reaches 53.7 months.

So far, little is known about the clinical course of asthma following withdrawal of the biologic treatment. In our study, 22 out of the 23 patients enrolled maintained good clinical control up to the last available evaluation, as confirmed by GINA and CASI scores which showed no statistically significant differences across the follow-up assessments. These results are consistent with the findings of Ferraro et al. (11), who analysed the first 12 months of follow-up after Omalizumab discontinuation. Moreover, sustained disease control after treatment discontinuation is supported by the reduction of equivalent fluticasone daily dose during the two-year follow-up (100 mcg/day at 24 months after discontinuation vs. 200 mcg/die at the baseline, *p* = 0.012). This finding, in line with previous published data (12), suggests persistent disease control, which allowed for a progressive tapering of maintenance ICS.

As for lung function after biologic withdrawal, we found normal values for FEV1, FEV1/FVC and FEF25-75 with no significant changes observed over the 24-months follow-up period. These findings confirm that lung function remains stable following biologic discontinuation. Previously published studies are consistent with our results and have similarly documented preserved respiratory function even after the discontinuation of biologic therapy (9–13).

Interestingly, our findings demonstrated that while clinical remission, defined as the absence of symptoms and exacerbations, was achieved in the majority of patients, pathophysiological remission, defined as normal lung function and absence of airway inflammation, was observed in only a small proportion of patients. These results highlight the need for a prolonged follow-up of patients in whom biologic therapy is discontinued, in order to monitor not only clinical symptoms but also the course of lung function and inflammation biomarkers.

Finally, although it is well established that biologic therapy leads to improvements in quality of life (18), it remains unclear whether this improved quality of life is maintained after discontinuation of biologic treatment. Our study demonstrated that both the total PAQLQ score and the scores of the three specific domains (symptoms, activities, emotions) remained stable over time. The absence of statistically significant differences in the timepoints evaluated compared to baseline suggests that a good quality of life is preserved even after the withdrawal of biologic therapy.

Although the general trend of asthma symptoms, lung function and quality of life strongly support a persistent good control of asthma in children and adolescents with severe asthma after biologic withdrawal, in this study we also tried to describe the characteristics of patients who did not maintain a perfect control over time. Analysing patients who were not fully controlled at 24-month follow-up, we found that they showed a significantly higher eosinophil count at T1 (3 months) and T3 (12 months) compared to the fully controlled group (T1 *p* = 0.045, T3 *p* = 0.02). A previous study showed that among patients who had discontinued Omalizumab therapy, a significant higher blood eosinophil count was found in patients who experienced exacerbations than in those who did not (19). Interestingly the only patient in whom treatment with Omalizumab had to be resumed nine months after discontinuation due to inadequate asthma control showed at T1 elevated FeNO levels (105 ppb), increased total serum IgE (812 KU/L) and blood eosinophil counts (220 cells/μl). Notably, IgE levels had increased by 230% compared to pre-treatment values, a percentage higher than the cohort average and closer to that reported by Vennera MDC et al. in patients who did not maintain good asthma control (median increase of 256%) (20). The role of eosinophils as potential predictors of suboptimal long-term asthma control, along with the biomarker trend observed in this patient who lost control after discontinuing therapy, jointly underscore the importance of monitoring biomarkers after biologic withdrawal.

## 5 Strengths and limitations

A major strength of this study lies in the novelty of the topic and the limited availability of existing data in this specific area, making our findings particularly valuable. In fact, showing persistent good clinical control after biologics discontinuation, our data suggest the possible disease modifying effect of this treatment in pediatric severe asthma. Moreover, our findings have potential implications for optimizing treatment duration, improving cost-effectiveness, and enhancing patient quality of life.

This study has some limitations. First of all, the retrospective nature of the study partially affected the completeness of the recorded variables, with some data lacking, mainly concerning biomarker measurements. Secondly, the small sample size and the small number of patients who completed the 24-month follow-up reduced the statistical power and generalizability of our findings, and prevented us from possible subgroup analyses. Specifically, we could not perform meaningful comparisons between different biologic treatments. As a result, most of our analysis report aggregated data from patients treated with various biologics, rather than analyzing them separately, which would have allowed a better understanding of whether different biologics are associated with distinct clinical courses and outcomes.

## 6 Conclusions

In conclusion our data demonstrate a persistent clinical and functional control in patients treated with biologics, even after the drug is discontinued. Over the 12–24 months after biologic discontinuation due to persistent good disease control, children exhibited no increase in asthma exacerbations, maintained good symptom control as assessed by GINA and CASI score, and preserved a good quality of life according to PAQLQ score. Only one adolescent resumed biologic therapy due to asthma worsening, suggesting that dependence on biologic therapy may be limited to a minority of children with severe allergic asthma. Our study underscores the importance of a close follow up, including assessment of clinical control, functional parameter and biomarkers, in children and adolescents in whom a biologic therapy is stopped to promptly identify signs associated with possible loss of control.

## Data Availability

The raw data supporting the conclusions of this article will be made available by the authors, without undue reservation.
